# Metformin Impact on Maternal and Infant Cardiometabolic Health (MIMICH), an open-label randomised controlled trial, and Metformin Impact on Maternal and Cardiometabolic Health After Pregnancy (MIMICH II)

**DOI:** 10.1186/s13063-025-09154-5

**Published:** 2025-10-17

**Authors:** Anya Amica-Carpenter, David Richard Cox, Yvonne Sylvestre, Stephen A. Roberts, Reena Perchard, Eleasha Calloway, Kerrie Richardson, Jenny Myers

**Affiliations:** University of Manchester, Manchester, UK

**Keywords:** Diabetes, Pregnancy, Metformin, RCT, Placental disease, FGR, SGA, Cardiometabolic health

## Abstract

**Background:**

Whilst women with diabetes are at risk of having a pregnancy complicated by foetal macrosomia, a small but important minority of women develop placental dysfunction leading to a small for gestational age infant (SGA < 10th centile) (20%) and/or the development of pre-eclampsia (12–18%) which may in some cases require preterm birth. In the UK, currently, metformin is offered as first line to treat diabetes during pregnancy, regardless of the risk of placental disease; however, the effect of metformin on placental function and foetal growth remains unclear. Metformin Impact on Maternal and Infant Cardiometabolic Health (MIMICH) and Metformin Impact on Maternal and Infant Cardiometabolic Health After Pregnancy (MIMICH II) aim to better understand the impact of metformin in the context of pregnancies at high risk of placental disease by evaluating the effect of metformin on foetal growth, placental function and maternal and infant cardiometabolic health.

**Methods:**

MIMICH is a randomised control trial (RCT) comparing routine treatment for diabetes, the standard of care, to withholding metformin (the intervention) during pregnancy, in women with type 2 diabetes or gestational diabetes (GDM) who have maternal or uteroplacental risk factors for placental disease in a target sample of 225 participants. Participants receive the recommended schedule of antenatal care, including serial biometry ultrasound with the addition of fractional thigh volume measurement. The primary outcome will be change in estimated weight z-score from second trimester to birth. The MIMICH II study will follow up the mothers and infants at 3–6 months and 12–15 months after pregnancy. Study procedures include anthropometric measurements from both mother and baby, data collection about medications, complications after birth and feeding, and maternal blood sampling. Infant echocardiography, blood pressure measurements and venepuncture will also be undertaken where possible.

**Discussion:**

Previous studies investigating hypoglycaemic agents in pregnancy have used birthweight as the primary outcome. In pregnancies where there are conflicting exposures influencing foetal growth and placental function (e.g. hypertension and hyperglycaemia), absolute birthweight is not sufficient to detect subtle, but clinically significant changes in foetal growth and placental function. To address this limitation, longitudinal measures of foetal growth will be used to quantify the deviation in foetal growth trajectory in the third trimester, using comparison with foetal growth prediction based on second trimester biometry from the same foetus.

**Trial registration:**

ISRCTN13866189. Registered on February 15, 2021.

**Supplementary Information:**

The online version contains supplementary material available at 10.1186/s13063-025-09154-5.

## Introduction

### Background {6a}

Amongst women* with poor cardiometabolic health embarking on pregnancy, hyperglycaemia is a very common feature. The prevalence of hyperglycaemia is increasing across Europe, alongside increasing rates of older maternal age, obesity and hypertension. Gestational diabetes (GDM) rates have recently been estimated at 5.4% (3.8–7.8) [1] and the prevalence of type 2 diabetes has doubled (0.5–1.1% between 2008 and 2012) [2]. As a result of the fact that the majority of women with hyperglycaemia have other cardiometabolic risk factors (family history, obesity, dyslipidaemia, hypertension), compared to healthy subjects these women have a three- to fourfold increased risk of pregnancy hypertension (7.4%) [3]. Rates of pre-eclampsia in women with type 2 diabetes have been reported to be as high as 31% [4] and women with a combination of diabetes and vascular disease are six times more likely to develop foetal growth restriction (FGR) [5].

Birth weight is a well-established factor for adult cardiometabolic disease. The short- and long-term impacts of prematurity and growth restriction for infants are much greater than of macrosomia [6]. Whilst foetal macrosomia is the common pathology in women with hyperglycaemia, an important minority (~ 3%) [5] will conversely develop severe placental disease leading to FGR requiring early preterm delivery to prevent stillbirth [7]. An additional 20% will have a small for gestational age (SGA, < 10th centile) baby and 12–18% will develop pre-eclampsia [3, 5].

*Throughout the protocol, in our references to women, we also encompass birthing people within this context.

#### Background: antenatal influence of metformin

Current practice in the UK is to offer metformin to all women with hyperglycaemia irrespective of potential risk factors for placental disease [8, 9], but the effect of metformin on placental function and foetal growth remains uncertain [10]. A recent Canadian RCT of women with type 2 diabetes demonstrated increased rates of SGA in women treated with metformin [11]. Another recent RCT comparing metformin and insulin in Spain found no difference in growth as a secondary outcome [12], and a meta-analysis of RCTs in 2024 found no difference in SGA rates between metformin and insulin treatment [13]. Notably, however, these studies are not looking primarily at growth restriction and do not consider the sub-set of patients with risk of placental dysfunction. In an animal model, metformin has been shown to accumulate in the foetus, demonstrated at delivery, and is linked to growth restriction [14].

The effect of metformin on cellular metabolism has not been fully explained and remains controversial; metformin has been shown to disrupt mitochondrial respiration [15, 16], alter redox status [17], impair tumour growth [18] and cellular proliferation under hypoxic and nutrient deficient conditions [19]. All of these actions suggest that metformin could negatively affect placental function particularly in the context of oxidative stress and hypoxia, although this has not been tested directly. Conversely, metformin has been demonstrated to have significant anti-inflammatory properties in the systemic vasculature [20, 21] and in trophoblast cell lines [22]. There is some circumstantial evidence from the clinical trials, which have used metformin to limit foetal growth in women with hyperglycaemia, that metformin reduces the risk of gestational hypertension [23, 24]. However, the effect on the rate of pre-eclampsia was not clear [25]. Meta-analysis by Wu et al. [13] showed no difference in gestational hypertension between metformin and insulin, but less pre-eclampsia in metformin treatment. Disagreement in the literature about these may reflect different disease aetiologies between some types of pre-eclampsia and gestational hypertension. The potential relationship between metformin and hypertensive disease is further highlighted by the finding that hypertensive disease correlates with the need for insulin treatment and metformin monotherapy failure [26]. The effect of metformin on both maternal cardiometabolic health and placental function therefore warrants further investigation so that this therapy can be targeted to the women who will benefit most [10].

#### Background: postnatal influence of metformin (MIMICH II)

To date, there is only limited evidence regarding the impact of in utero metformin exposure on childhood metabolic health. Mouse studies investigating prenatal exposure to metformin have reported opposing effects on glucose tolerance and insulin sensitivity. In a study by Gregg et al. [27], improved glucose tolerance was demonstrated in male offspring exposed to metformin in utero. In contrast, in a study by Salomäki et al. [28], metformin-exposed offspring were lighter at birth but demonstrated excessive weight gain and impaired glucose tolerance when fed a high-fat diet after weaning. The limited available human studies have demonstrated that children exposed to metformin in utero are heavier at 12 months [29], taller and heavier at 18 months [30] and have increased subcutaneous fat deposition at the age of 2 (Metformin in GDM (MIG) Trial) [31]. Children from the MIG trial have now been followed up to age 7 (Adelaide) and 9 (Auckland) [32]. Whilst there were no significant differences in the age 7 children from Adelaide, the children from Auckland aged 9 were slightly larger measured by weight, arm/waist circumferences and waist:height ratio (*p* < 0.05). A recent meta-analysis has synthesised the available childhood follow-up data and from pregnancy studies where metformin was prescribed to women with polycystic ovarian syndrome (PCOS) and uncomplicated GDM (338 children exposed to metformin and 346 not exposed) [33]. Overall, metformin-exposed children were heavier than controls (standard mean difference 0.26 [0.11–0.41], *p* = 0.0008) at 2 years.

The MiTy trial, which showed an association with SGA in the metformin-treated group, recently published data about the infants of these mothers at 2 years and found their anthropometric measures were similar between groups at the 2-year endpoint, but that metformin-exposed male infants had a different growth trajectory, and that BMI was higher from 6 to 24 months [34]. Postnatal catch-up or accelerated growth often defined as a delta weight SD score > 0.5 or 0.67 has been consistently associated with insulin resistance and metabolic syndrome-related disorders (e.g. type 2 diabetes) in later life [35–37]. Accelerated growth during early postnatal life, irrespective of birth weight, has been shown to associate with changes in β-cell function at 12 months [38, 39] and appears to be particularly important for metabolic health in later life [40]. It is unclear from existing studies whether metformin exposure in utero affects postnatal catch-up and therefore impacts future cardiometabolic health in later life. Of note, infants exposed to metformin in the EMPOWaR study [41], a randomised study of obese pregnant women, were thinner (lower ponderal index) at birth, which, if associated with accelerated postnatal weight gain (akin to the mouse studies), could confer an increased cardiometabolic risk to the offspring [42].

#### Rationale

Given the uncertainty regarding the potential benefits (improvement in maternal metabolic health and reduction in hypertensive disease), but potential negative effects on placental function (reduction in foetal growth and antiproliferative cellular actions), highlighted above and by a recent expert review [10], a trial of metformin in women with hyperglycaemia and risk factors for placental disease is justified. In addition, it is essential that effects on offspring are also investigated, and doing so in this population allows for antenatal foetal growth trajectory and postnatal infant growth trajectory to be connected.

As it is routine clinical practice to offer metformin to women with hyperglycaemia, the intervention in this study will be to withhold metformin and manage hyperglycaemia with diet/insulin alone. A randomised study is essential to avoid treatment-selection bias, and the study is open-label to aid the ongoing management of diabetes. No previous studies have investigated the effect of metformin on placental function, foetal growth and maternal cardiometabolic health in women with hyperglycaemia and concurrent risk factors for placental disease. This study is made possible through access to a large population of high-risk pregnancies across Manchester (3 hospital sites serving 18,000 births per year) and the specialist translational clinic infrastructure which incorporates 3D volumetric ultrasound foetal growth assessment.

### Objectives {7}

#### Primary objectives

- To evaluate if withholding treatment with metformin in women, with type 2 diabetes or gestational diabetes (GDM) pregnancy AND risk factors for placental disease, affects foetal growth
- To determine what impact metformin has on early postnatal growth and metabolic function, in infants born to women with poor cardiometabolic health

#### Secondary objectives

- To investigate the effect of metformin treatment on placental function and measures of maternal cardiometabolic health
- To determine the impact of metformin on early postnatal growth and metabolic function, in infants born to women with poor cardiometabolic health
- To determine if maternal exposure to metformin during pregnancy improves maternal cardiometabolic health status 1 year after pregnancy

### Trial design {8}

MIMICH is a phase III, two-arm, single-centre, open-label pragmatic randomised controlled trial (RCT). The study framework aims to assess the superiority of withholding metformin in otherwise standard pregnancy diabetes care, determined by foetal growth trajectory and placental function. Randomisation will take place in a 1:1 ratio. Following the RCT, participants who provide consent will be enrolled in an observational cohort study assessing postnatal maternal and infant cardiometabolic health, with follow-up visits at 3–6 months and again at 12–48 months postpartum.

## Methods: participants, interventions and outcomes

### Trial setting {9}

The study is conducted within Manchester University NHS Foundation Trust (MFT) (single Hospital Trust). Women will initially be identified in antenatal clinics at three hospital sites within MFT (St Mary’s Oxford Rd, St Mary’s Wythenshawe, North Manchester General Hospital) and provided with information regarding the study. Confirmation of eligibility, consent and all study visits will take place within the Maternal & Fetal Health Research Centre, St Mary’s Oxford Rd. Pregnancy outcome data will be collected following review of clinical records by study midwives within MFHRC with assistance from CRN research staff at each of the identification hospital sites. Participation in the postnatal follow-up study will be offered to participants who consent to be contacted about future research. All procedures including screening, consent and study visits will take place at St Mary’s Hospital MFT by a delegated member of the research team.

Participants will continue to receive the standard pathway of care throughout the course of the trial, with the exception of the omission of metformin therapy for those participants randomised to the intervention arm.

### Eligibility criteria {10}

#### Inclusion criteria

1. Singleton pregnancy between 6^+0^ and 30^+0^ weeks’ gestation inclusive
2. Aged 18 years or over and willing and able to give informed consent
3. Diagnosis of diabetes in pregnancy
  - Type 2 diabetes diagnosed before pregnancy OR
  - Type 2/GDM diagnosed < 24 weeks’ gestation: abnormal glucose tolerance test^a^, abnormal HBGM^b^ and/or HbA1C ≥ 41 mmol/L OR
  - GDM (diagnosed 24–30 weeks): HbA1C ≥ 41 mmol/L and/or abnormal glucose tolerance test^a^
4. Abnormal HBGM.^b^ (≤ 30 weeks)
5. Presence of at least one risk factor for placental disease
  - BP ≥ 130 and/or ≥ 80 mmHg (clinic blood pressure)
  - Pulse wave velocity ≥ 9 m/s
  - Age ≥ 35 years
  - Nulliparous
  - Pre-eclampsia and/or small for gestational age (< 10th centile) in a prior pregnancy
  - Current SGA (EFW < 10.th centile)
  - Mean uterine artery PI ≥ 95th centile^c^
  - Placental growth factor < 10th centile^c^
6. EFW ≤ 50th centile (if ≥ 22 weeks)^c,d^

^a^Abnormal glucose tolerance test: fasting glucose ≥ 5.3 mmol/L and/or 2 h ≥ 8.5 mmol/L.

^b^Abnormal home blood glucose monitoring (HBGM): 3 abnormal readings (fasting blood glucose ≥ 5.3 mmol/L, 1 h post meal ≥ 7.8 mmol) in 1 week.

^c^To be confirmed/measured at the baseline visit.

^d^WHO population centiles [43].

#### Exclusion criteria

1. Medical contraindication to metformin
2. Known diagnosis of type 1 diabetes
3. Multifoetal pregnancy
4. Prior pregnancy complicated by shoulder dystocia
5. Known foetal abnormality identified prior to consent
6. Any neonate confirmed to have a serious congenital or chromosomal abnormality or any congenital abnormality which may impact postnatal growth will be excluded from MIMICH II

### Eligibility of those performing interventions {10}

Study instruments and procedures are implemented by the team providing clinical care to the participants in the consultant-led clinic. This comprises of research midwives, dietitians, diabetic and specialist nurses/midwives with the relevant professional degrees and advanced training to fulfil their clinical role, locally trained research practitioners, and obstetricians/endocrinologists medically qualified with higher speciality postgraduate training.

### Who will take informed consent? {26a}

Women who meet the inclusion criteria are identified and approached by members of the direct clinical care team or the antenatal diabetes team (who are integrated within the direct clinical care team) at antenatal clinics. Potential participants are provided with information on the study, both verbally from a member of the research team and written via the participant information leaflet. Participants are given the opportunity to ask any questions they may have and then provided with appropriate time to decide whether they wish to participate. Eligibility is confirmed by a medically qualified individual. Consent is obtained by an appropriately trained healthcare professional (who is Good Clinical Practice (GCP) trained and designated to take consent by the site principal investigator). Study participation and prescription information is documented and communicated as per usual care.

### Additional consent provisions for collection and use of participant data and biological specimens {26b}

The consent forms include statements confirming permission for data collected during the study to be accessed by members of the maternal and foetal health centre, who may not be directly involved in care and/or members of the university or regulatory bodies where relevant to the research, and that collected data will be anonymised and included in publications related to the trial.

Additionally, the MIMICH consent form also includes statements confirming the collection of blood and placenta samples and their storage and distribution within the maternal and foetal health research centre.

Additional consent is obtained for participation in the follow-up postnatal study (MIMICH II).

The MIMICH II consent forms contain statements relating to the collection and storage of infant blood samples and to the collection and storage of maternal blood samples.

## Interventions

### Explanation for the choice of comparators {6b}

The comparators are diabetes treatment with or without metformin. Dietary and lifestyle modification with insulin treatment where required are standard components of treatment that will be provided to all participants in both arms, with the only difference being the use of metformin. Withholding metformin and normalising blood glucose levels with insulin alone will enable the impact of metformin on foetal growth and maternal and infant cardiometabolic health to be investigated.

### Intervention description {11a}

Standard diabetes care follows the recommendations in NICE guidance NG3: ‘Diabetes in pregnancy: management from preconception to the postnatal period’. Home blood glucose monitoring (HBGM) may be either intermittent capillary glucose measurement or continuous glucose monitoring (CGM). Glucose targets, as per NICE, are fasting, 5.3 mmol/L and 1 h post-prandial, 7.8 mmol/L. Metformin is commenced where these targets are not met with diet and lifestyle advice/modification (in line with NICE guidance and guided by dietitian referral). Metformin dose is titrated to maternal tolerance as well as glycaemic control, starting at 500 mg twice daily with meals, increased every 3–5 days up to a maximum of 2500 mg daily in divided doses. Insulin is introduced where glucose targets are not met having reached the maximum tolerated dose of metformin. Insulin prescription is tailored to individual glycaemic profile using a long-acting (detemir or isophane) or rapid-acting (aspart) insulin, in isolation or combination. Insulin dose changes will be recommended to participants at a minimum of 3-day intervals according to HBGM as per standard clinical care. Diabetes treatment is supervised by a team of specialist midwives, obstetricians and diabetologists.

### Criteria for discontinuing or modifying allocated interventions {11b}

Usual clinical care will be provided (following local and NICE guidelines), and medication will be titrated to glucose levels; no trial-specific treatment modifications are planned. Stopping or switching diabetes treatments is a common part of usual clinical care in pregnancy, and in order to maintain safety, it is anticipated that some participants will need to take metformin in the intervention arm. In addition, some participants allocated to usual care will not be able to tolerate metformin. Failure to tolerate metformin (usual care arm) will not be reported as an adverse event unless related to a reportable serious adverse event. All modifications to treatment allocation will be documented and included as secondary outcomes. A participant can continue in the study after discontinuation of treatment.

### Strategies to improve adherence to interventions {11c}

As a pragmatic trial, standard clinical advice on adherence to diabetes treatment will be advised and encouraged at every visit with no additional specific trial advice given. The daily dose of metformin (0–2.5 g), long- and short-acting insulins taken over the preceding week will be recorded at each study visit.

### Relevant concomitant care permitted or prohibited during the trial {11d}

As the MIMICH trial is embedded within usual diabetes in pregnancy care, no concomitant care is prohibited for either arm of the study. Enrolment in observational studies is permitted, and co-enrolment in other intervention studies will be considered and discussed by the trial management team.

### Provisions for post-trial care {30}

Involvement in the MIMICH trial will cease following discharge from hospital after birth; all participants will receive standard postnatal care including management and treatment of diabetes. For eligible participants who agree to take part, additional follow-up visits will be offered as part of the MIMICH II study.

### Outcomes {12}

#### MIMICH

Outcomes will be recorded in the electronic case report form, hosted on REDCap. Outcomes will be collected from baseline to maternal discharge post-birth. Neonatal outcomes will be recorded up to 28 days after birth.

Primary outcome:

Third trimester foetal growth velocity, assessed by change in foetal growth z-score between 20 and 29 weeks’ gestation (average) and birth.

At each scan, the estimated foetal weight (EFW) in grams will be calculated using 2D biometry and fractional thigh volume (TVol) measurements using a standard formula [44] and converted to a z-score [43]. The delta change in z-score between the average z-score of scans performed between 20 and 29 weeks and the birthweight z-score will be calculated.

Secondary outcomes:

During pregnancy, secondary outcomes will be reported as per standard for pregnancy intervention trials. Secondary foetal growth and birthweight analyses will be assessed according to treatment group in line with the primary outcome analysis. Outcomes relating to blood glucose control (as an indicator of treatment effect) will similarly be compared between groups, as will blood pressure and hypertensive complications. Reporting and analysis of secondary outcomes will be described in full in the statistical analysis plan; to avoid issues of multiple testing, not all secondary outcomes will be compared between treatment groups. Secondary outcomes will be assessed using adjusted linear regression for continuous outcomes and chi-squared for categorical outcomes; *p* values > 0.01 and < 0.05 will be interpreted with caution. Where appropriate, outcomes will be presented with a treatment effect and confidence intervals; other outcomes will be presented with summary statistics only.

Prespecified secondary outcomes:

Adherence/acceptability:

- Number of missed doses—calculated as an average over pregnancy from the number of missed doses reported in the 7 days prior to each study visit (adjusted for number of study visits)
- Number of women unable to comply with allocated treatment (cross over)
- Undesirable effects of allocated treatment (number of women reporting undesirable effects associated with metformin and/or insulin/number of women receiving metformin or insulin treatment)
- Treatment satisfaction with allocated medication regime (questionnaire)
- Acceptability of allocated medication regime (questionnaire)

Secondary foetal growth research outcomes:

- Distribution of foetal growth z-scores between treatment groups and within prespecified subgroups
- Comparison of foetal growth z-scores (conventional Hadlock formula EFW without TVol measurement) between treatment groups and within prespecified subgroups
- Number of small and large for gestational age infants (defined using birthweight z-score) derived from the WHO birthweight centiles [43]

Standard secondary maternal outcomes:

- Gestational weight gain (difference between baseline and 30–34-week visit weight adjusted for number of weeks)
- Episodes of severe hypoglycaemia (blood glucose < 3 mmol/L)—total number reported over the treatment duration reported at each study visit
- Mean (fasting and 1 h post meal) daily glucose and % time in target—captured from HBGM and/or CGM sensors at each study visit and summarised for each trimester
- Maximum achieved dose of metformin (standard care arm only)
- Total insulin dose (units/kg/day) at final study visit (short- and long-acting insulin reported separately)
- Mean change in insulin dose (units/day) from baseline to last study visit
- Need for antihypertensive therapy—study visits and pregnancy outcome case note review
- Pre-eclampsia (defined according to ISSHP guidelines)—study visits and pregnancy outcome case note review
- Indicated delivery (induction of labour or pre-labour rupture of membranes (PROM) with stimulation of labour or pre-labour caesarean section)—pregnancy outcome case note review
- Mode of onset of birth (spontaneous, induction of labour, PROM with stimulation of labour, pre-labour caesarean section)—pregnancy outcome case note review
- Indication for onset of birth—pregnancy outcome case note review
- Mode of birth—pregnancy outcome case note review
- Postpartum haemorrhage (blood loss > 1000 mL)—pregnancy outcome case note review
- Shoulder dystocia—pregnancy outcome case note review
- Total number of postnatal hospital inpatient days—pregnancy outcome case note review

Neonatal outcomes—clinical birth outcomes obtained from case note review:

- Foetal loss (prior to 24 weeks’ gestation and from 24 + 0 weeks’ gestation (stillbirth))
- Known early neonatal death (up to 7 days from birth)
- Known late neonatal death (between 7 and up to 28 days from birth)
- Gestational age at birth
- Birthweight, birthweight centile, birthweight z-score
- Neonatal unit admission (separation of baby from mother), principal recorded indication for neonatal unit admission and length of stay in neonatal unit (and level of care)
- Apgar score (5 min)
- Umbilical arterial pH at birth
- Need for respiratory support and type of respiratory support needed
- Lowest blood glucose measurement and need for treatment for neonatal hypoglycaemia
- Neonatal seizures, intracranial haemorrhage, necrotising enterocolitis
- Breastfeeding at discharge

Exploratory outcomes—additional research outcomes:

- Longitudinal changes in angiogenic markers (sFlt, PlGF)—measured in research blood samples (batch analysis end of study)
- Maternal skinfold measurements (delta change from baseline visit adjusted for gestation)
- HOMA-IR (measured at 30–34 weeks ± 2 weeks)—measured in research blood samples (real time in the biochemistry lab)
- Ponderal index (foetal weight in grams × 100/(foetal length in centimetres)
- Neonatal measurements within 72 h of birth (crown-heel lengths, weight, head/abdominal circumferences, biceps, triceps, subscapular skinfold thicknesses)
- Erythropoietin (marker of intrauterine stress) [45, 46]

#### Outcomes for MIMICH II

Primary infant outcome: Difference in the delta (birth to visit 2) weight SD score between infants following maternal treatment with or without metformin

Infant secondary outcomes:

- Metabolic health indices including biochemical (insulin, glucose, lipid profile, leptin, adiponectin)
- Anthropometric measurements (crown-rump, crown-heel lengths; weight; arm, thigh, head, abdominal circumferences; biceps, triceps, subscapular skinfold thicknesses) expressed as z-scores using WHO charts where applicable
- Cardiac measurements including systolic and diastolic blood pressure and echocardiogram (left ventricular dimensions, diastolic function and systolic function)
- Transcriptomics and metabolomics are collected from infant and mother and differentially expressed genes and metabolites will be determined between metformin-exposed and non-exposed infants

Maternal secondary outcomes: Postnatal weight change (from booking to late pregnancy), adiposity (waist:hip ratio, biceps, triceps, subscapular skinfold thicknesses), cardiometabolic health measurements (insulin resistance, lipids, hsCRP, inflammatory cytokines, BP)

#### Adverse events

Adverse event recorded (number of women and number of adverse events) during treatment only. All AEs and SAEs identified will be recorded in the trial database and will be reviewed and signed by the PI. The trial sponsor will also be made aware of any SAEs that occur on trial and will be sent copies of the completed SAE forms.

As postnatal follow-up visits will not require treatment to be taken, any adverse events occurring during this time will not be recorded in the trial database.

If any abnormality is found in the bloods, blood pressure or echocardiograms at any point during active treatment or postnatal follow-up, this will be explained to the participant and we will inform their GP or refer to the appropriate clinical team depending on the clinical significance of the finding.

### Participant timeline {13}

**Fig. 1:**
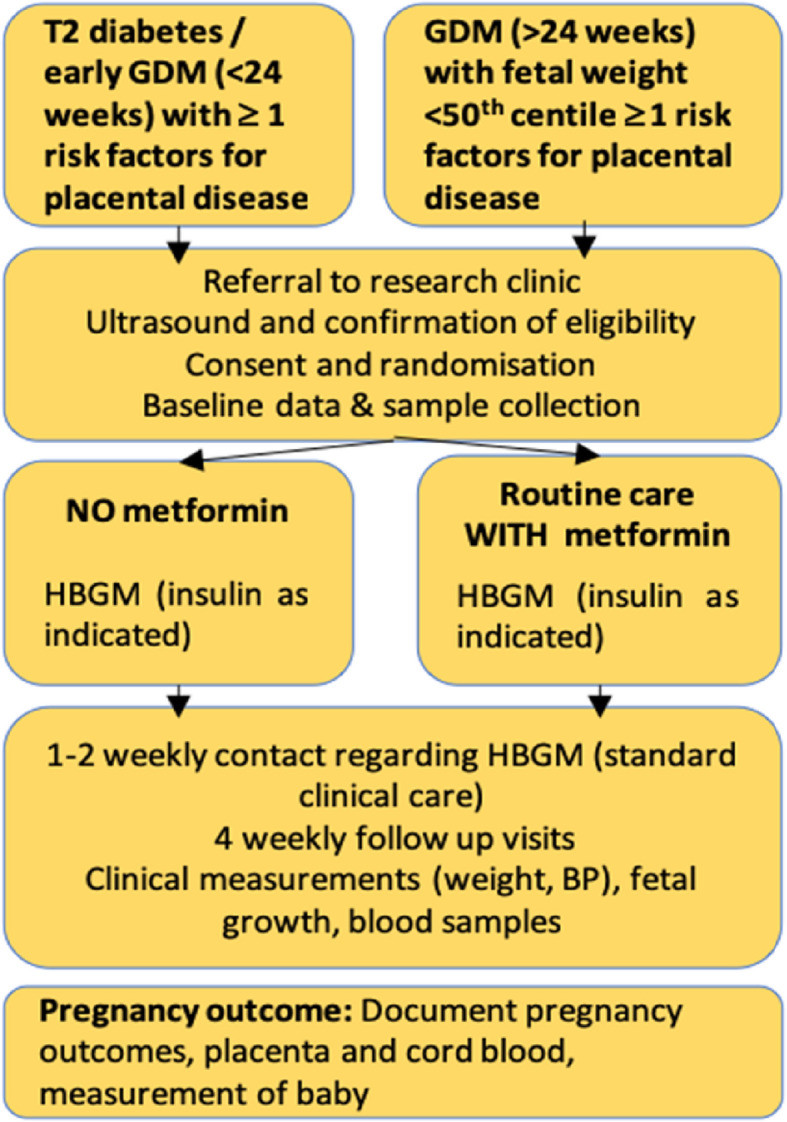
Timeline of protocol for MIMICH participants

The participant timeline for active treatment is presented in Fig. 1 and Table 1. As MIMICH is a pragmatic, phase III trial to refine the provision of routine antenatal care, no additional visits are required outside of normal care.

**Table 1:** Schedule of visits and trial procedures for MIMICH

Procedures	Participant identification antenatal clinics	Baseline visit	Intervention period (study visits every 4 ± 2 weeks)	Fasting visit (30–34 weeks ± 2 weeks)	At/after birth
Routine antenatal visit	x	x	x	x	
Eligibility check	x	x			
Written informed consent		x			
Randomisation		x			
Collection of baseline characteristics (demographics, obstetric and medical history, medication)		x			
Confirmation of willingness to continue intervention			x	x	
Study visits every 4 ± 2 weeks (including ultrasound scan, BP, weight)		x	x	x	
Skinfold measurements		x		x	
Adherence with treatment: dose of insulin/metformin and self-reported number of missed doses in previous 7 days		x	x	x	x
Summary of glycaemic control and number episodes of hypoglycaemia (preceding 7 days)		x	x	x	x
Fasting blood samples (30–34 weeks only)				x	
Acceptability questionnaire completion [hard copy]				x	
Review of pregnancy complications/events			x	x	x
Research blood samples		x	x	x	x
Safety reporting			x	x	x
Pregnancy outcome/case note review					x
Collection of placenta and cord blood					x
Measurement of infant (within 5 days of birth)					x

**Fig. 2:**
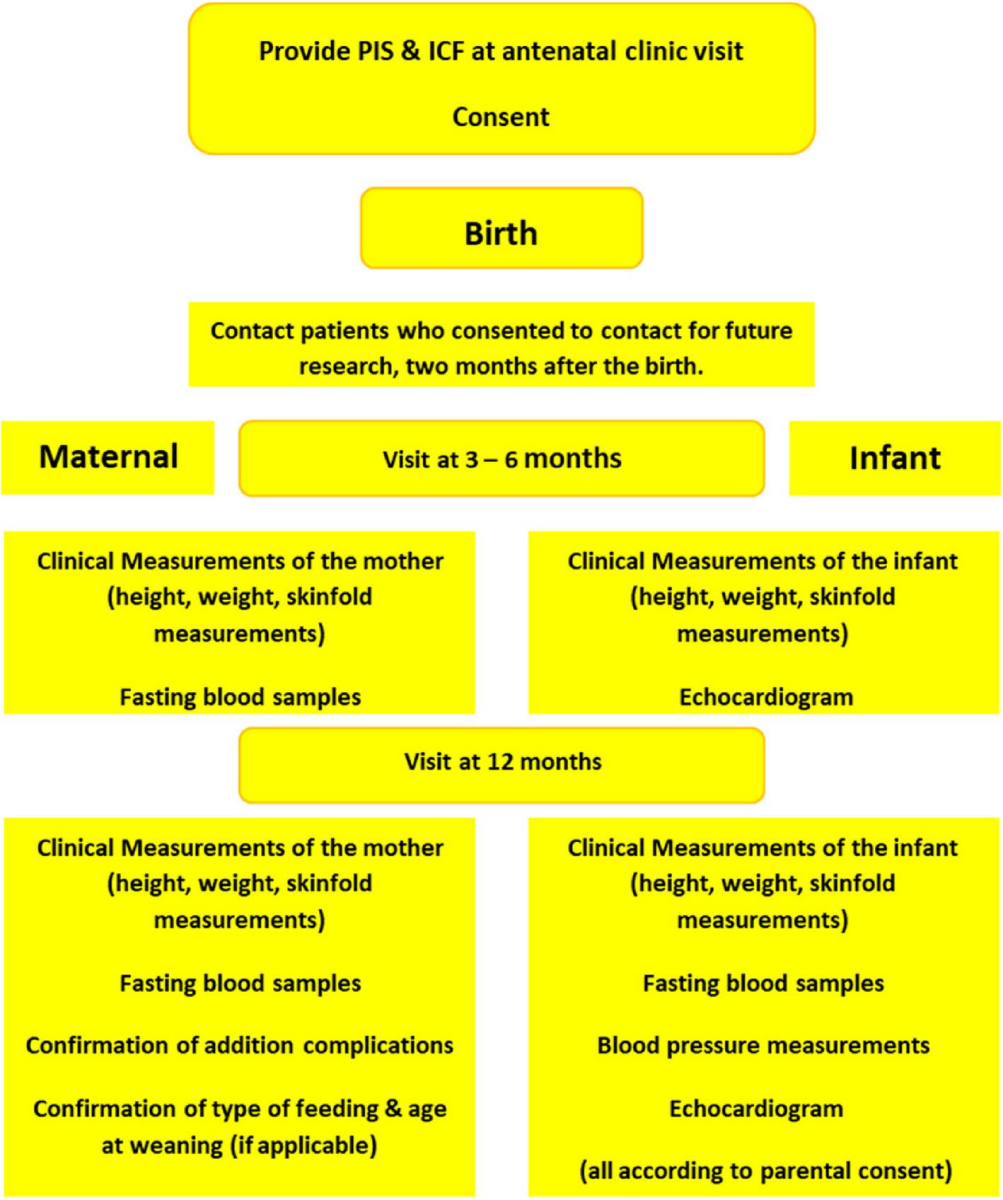
Timeline of protocol for MIMICH II participants

Figure 2 and Table 2 depict the timelines for postnatal follow-up. As MIMICH II is concerned with postnatal care, all study visits will fall outside of routine care.

**Table 2:** Schedule of visits and trial procedures for MIMICH II

Procedure	MIMICH study visit	Birth	3–6 months postnatal visit	12 months postnatal visit
Consent	X			
Collection of maternal and neonatal outcome data		X		
Collection of clinical measurements			X	X
Maternal fasting blood sample			X	X
Infant fasting blood sample				X
Completion of study questionnaire			X	
Confirmation of willingness to continue			X	
Confirmation of additional complications				X
Confirmation of infant feeding type and age at weaning				X
Infant echocardiogram			X	X
Infant blood pressure				X

### Sample size considerations {14}

The primary outcome is a change in foetal growth velocity in the third trimester. Our group has pioneered the use of volumetric ultrasound techniques to improve the detection of foetal growth abnormalities [47, 48]. Previous studies investigating hypoglycaemic agents in pregnancy have used birthweight as the primary outcome, which provides only a crude estimate of foetal growth. In pregnancies where there are conflicting exposures influencing foetal growth and placental function (e.g. hypertension and hyperglycaemia), absolute birthweight is not sufficient to detect subtle, but clinically significant changes in foetal growth and placental function. To address this limitation, in this study, longitudinal measures of foetal growth will be used to quantify the deviation in foetal growth in the third trimester. This will be derived from the delta z-score in estimated foetal weight (using the modified EFW TVol formula [44]) over the third trimester using WHO foetal growth charts [43]. This will provide a more sensitive assessment of the effect of metformin on foetal growth than birthweight alone; the use of a continuous measure as a primary outcome also makes the study more efficient as it requires a smaller sample size. In order to verify the primary outcome, the eCRF will capture the TVol measurement and the EFW at each scan as well as recording the 2nd trimester and 3rd trimester deviation scores. The source data (scan report) will verify the 2D biometry.

In a comparison of women with type 2 diabetes treated with metformin recruited to the VELOCITY study, the mean delta z-score between the second trimester and birth was − 0.51 (SD 1.1) for women with a placental disease risk factor (*n* = 34) and 0.30 (SD 0.9) in women without a risk factor (*n* = 45). A sample size of 190 women (95 in each group) will provide 80% power to detect a clinically significant difference in delta z-scores of 0.41 (SD 1.0) between the treatment groups; this difference would reflect a rightward shift in the birthweight distribution in women in whom metformin was withheld to a mean of − 0.1.

An adjusted analysis of the effect of the intervention (withholding metformin) on foetal growth will include covariates (mean daily glucose, time in target) which are known to influence foetal growth to improve the precision of the treatment effect as appropriate. Change in foetal weight z-score provides a continuous measure of late pregnancy growth trajectory derived from early foetal biometry and 3D volumetric measurements and therefore the foetus is its own control. Importantly, this removes the need to adjust for infant sex. Furthermore, as the measurement of foetal weight z-scores using 3D foetal thigh volumes, and therefore calculation of delta z-scores is done after birth, the risk of bias is minimised. In the MiTy trial [11], a comparison of metformin vs no metformin in unselected women with type 2 diabetes, the difference in birthweight z-score was − 0.28 (− 0.45 to − 0.10).

Recruitment figures have been based on attendances to the Manchester Antenatal Vascular Service (MAViS) clinic, which provides care for women with cardiometabolic disease, and the diabetes service across Manchester Foundation Trust. Allowing for the fact that the proposed study will draw from a population of 16,500 pregnancies per annum, eligible women will include those with type 2 diabetes (*n* ≈ 108) and women who develop GDM with additional risk factors for placental disease (*n* ≈ 420). A recruitment rate of 50% would yield an available cohort of approximately 225 women (10% dropout) over a 36-month time frame. Based on figures from the MAViS cohort, 20% would be expected to develop a small for gestational age (SGA < 10th centile) and 12–18% pre-eclampsia.

The primary outcome for the postnatal follow-up study will be weight gain trajectory, determined as change in weight z-score, between birth and 12 months of age. Assuming a population z-score weight gain of 0.08–0.12 (SD 0.9) [37, 38] at 3–6 and 12 months, *n* = 39 (*β* = 0.8) or *n* = 70 (*β* = 0.9) per group will be required to show a 0.5 difference in z-score between groups (retention 35–63%). Excessive infant weight gain (> 0.67 SD, representative of the width of each percentile band on standard growth charts (e.g. 2nd–9th, 9th–25th percentile [8]) in the first year of life is strongly associated with an adverse adult cardiometabolic risk profile [36, 37]. Previous studies have demonstrated significant differences in adult cardiometabolic indices between groups of infants with rapid vs slow weight gain in the first 3–6 months after birth [49] using data from 65 and 22 adults, respectively. A conservative 0.5 change in z-score between metformin-exposed and non-exposed infants (at each time point) will therefore represent a robust and important clinical difference. Covariates (maternal glycaemic control, infant feeding, birthweight z-score and infant height) likely to be associated with the primary outcome will be included in the adjusted analysis to improve the precision of the treatment effect as appropriate. This will be balanced against the loss of effective sample size through use of degrees of freedom following inclusion of covariates [50].

### Recruitment {15}

The recruitment window for the study will be 36 months from the point the trial opens. Women are initially identified in the antenatal care setting of St Mary’s Hospital MFT, St Mary’s Hospital Wythenshawe and North Manchester General Hospital. Eligible participants are provided with information on the study and given sufficient time to consider participation. Those interested in the study are invited to attend an enrolment and consent visit at St Mary’s Hospital MFT. Eligible participants are invited to take part in MIMICH II. Strategies to ensure recruitment targets are met include offering incentives such as continuity of care from the trial team throughout pregnancy, 4D ultrasound scans and gift vouchers. Additionally, participants of MIMICH II will receive MIMICH branded baby grows, have their travel expenses covered and will receive postnatal care from the same trial team who performed their antenatal visits.

## Assignment of interventions: allocation

Participants will be randomised via the online gateway ‘randomisation system’ hosted by the independent provider Sealed Envelope.

### MIMICH sequence generation {16a}

The sequence will be computer generated within the Sealed Envelope platform. Participants will be randomised (1:1) using minimisation with a random element controlling for:

- Type of diabetes (diagnosed < 24 weeks’ gestation, diagnosed > 24 weeks’ gestation)
- Risk factor for placental disease (uteroplacental or maternal risk factor)
- Gestational age (6 + 0 to 11 + 6 weeks, 12 + 0 to 23 + 6 weeks, 24 + 0 to 30 + 0 weeks)

The sequence will be computer generated. Participants will be randomised (1:1) using minimisation with a random element controlling for:

- Type of diabetes (diagnosed < 24 weeks’ gestation, diagnosed > 24 weeks’ gestation)
- Risk factor for placental disease (uteroplacental or maternal risk factor)
- Gestational age (6 + 0 to 11 + 6 weeks, 12 + 0 to 23 + 6 weeks, 24 + 0 to 30 + 0 weeks)

For each new participant, the algorithm calculates the imbalance that would result from assigning them to each treatment arm. The arm that minimises imbalance is selected with high probability (default 80%). With the remaining probability (20%), allocation is made to the alternative arm. If both arms would produce equal balance, allocation is determined with equal probability (50:50).

This approach maintains a close balance between groups across important prognostic factors, whilst the random element preserves allocation unpredictability. The randomisation sequence is generated and maintained centrally within the Sealed Envelope system, ensuring full allocation concealment from investigators and participants until the point of assignment.

### Concealment mechanism {16b}

The randomisation process is centrally and independently managed by Sealed Envelope, which ensures concealment of the allocation sequence until the time of intervention allocation.

### Implementation {16c}

The responsibility for enrolling participants into the trial lies with appropriately trained healthcare professionals at the site, who will have to confirm the eligibility criteria before they enter the minimisation factors into the online randomisation system (sealed envelope) to randomise participants. Using minimisation with a random element, the system will allocate the treatment, and users will be notified of the assigned treatment allocation for the participant. Allocation concealment will be ensured, as the service will not release the randomisation code until the patient has been recruited into the trial, which takes place after all baseline measurements have been completed.

### Blinding {17a and 17b}

To ensure women are effectively and safely treated during the trial with a focus on normalising blood glucose levels, participants, clinicians or members of the trial management team are not blinded to participant allocation and therefore no unblinding procedures are required. Data analysts (analysis of 3D thigh volumes) are blinded to treatment allocation.

### Data collection and management {18a}

Plans for assessment and collection of trial outcomes are presented in the Outcomes section above. Before commencing recruitment, all trial team members receive training on the study designs, protocol procedures (within the Site Initiation Visit) and data collection procedures (REDCap database training). All members of the site research team are expected to work in accordance with the GCP guideline and are required to provide proof of their GCP training and relevant experience.

Baseline information such as demographic data and pre-pregnancy health information, study visit data and outcome data are captured during study visits and from health records notes where clinical data is routinely collected during the antenatal period, at birth and up to hospital discharge.

Research specific data such as skinfold thickness information, treatment adherence in the seven days prior to the visit, current medications and baby measurements at birth and postnatally will be captured though study-specific source data collection forms and recorded contemporaneously during each study visit.

Instruments for data collection related to the trial include ultrasound machines, biochemical assays and glucose monitoring, and these are summarised in Appendix A.

Participant satisfaction and acceptability of trial intervention is captured in an unvalidated trial-specific questionnaire (Appendix B).

For participants who are ineligible for post-trial follow-up, outcomes are captured up to primary hospital discharge for each of the women or baby post-birth, and 28 days post-birth for neonates. For those who continue in the MIMICH II study, outcomes are collected up to 12–48 months postnatally for both the mother and baby. The case report forms for both MIMICH and MIMICH II have been created within the REDCap database hosted by the University of Manchester. PDFs of all study instruments have been downloaded and filed in the investigator site file (ISF) and trial master files (TMF).

Data monitoring procedures include the use of range control within data entry fields, gestational age calculations embedded in date fields and verification of all entered data by an independent data monitor; all data is verified against case report forms and case note review, with queries actioned and logged using REDCap functionality.

### Plans to promote participant retention and complete follow-up {18b}

All recruits will receive continuity of care in the specialist research clinic to encourage participation and retention. Confirmation of a willingness to continue in the trial will be confirmed at each clinical/study visit. Participants who choose to end their participation in the MIMICH trial but consent to the collection of birth and outcome data will be classed as discontinuations. In this case, no further data during antenatal care will be collected, but maternal, neonatal and birth details will be collected. Participants who end their participation and decline collection of their birth and outcome data will be classed as withdrawals. In this case, all data collection from the point of withdrawal will cease. Participants who discontinue or withdraw from MIMICH will not be eligible for MIMICH II. Participants who deviate from their allocated treatment arm will continue in the study, with treatment exposure recorded at each visit.

### Data management {19}

Trial data are collected and stored according to GCP guidelines. Processes to facilitate the accuracy of the trial data are detailed in the data management plan. Coding and validation are agreed upon between the trial manager and statistician, and the study database is signed off once the implementation of these has been assured. Only minimal identifiers (e.g. initials, date of birth, last four digits of NHS numbers and postcodes) will be included in the trial database. All paper records will be kept in locked filing cabinets, in a secure office during the trial. Access to partially anonymised trial data is controlled by the CI, with access approved by the REDCap data administrator. Electronic and hard copy data will be retained in line with the University of Manchester’s archiving standard operating practice (SOP).

### Confidentiality {27}

Personal information recorded is regarded as strictly confidential and handled and stored following the Data Protection Act 2018 (and subsequent amendments). Women are always identified using their unique study number. No information by which a woman may be identified is disclosed to any third party other than those directly involved in the treatment of the participant.

### Plans for collection, laboratory evaluation and storage of biological specimens for genetic or molecular analysis in this trial/future use {33}

Blood samples will be collected with permission from participants at every study visit. Where possible, placentas will be collected after birth. Samples will be stored in the Maternal and Fetal Health research laboratory for the duration of the trial, after which time they will be transferred to the Tommy’s National Biobank if consented to by the participants. Samples will be destroyed after the trial has ended if biobank storage is declined.

## Statistical methods

### Statistical methods for primary and secondary outcomes {20a}

The statistical analysis will be conducted according to the approved statistical analysis plan. Baseline characteristics of the trial population will be presented as mean and standard deviation (or median and interquartile range as appropriate). The main analysis will be an intention to treat analysis for the primary and secondary outcomes. For the primary outcome, the average z-score for estimated foetal weight (calculated using the modified EFW formula which incorporates TVol [44]) performed between 20 and 29 weeks will be calculated. This will be 1–3 scans per woman. The delta between the birthweight z-score and the 20–29-week z-score will be calculated. Delta z-scores will be compared between treatment groups using linear regression models adjusted for baseline z-score and minimisation variables (these will be confirmed in the final statistical analysis plan). All secondary outcomes will be reported as described in the outcomes section and analysed in accordance with the prespecified statistical analysis plan.

Missing data for baseline prognostic factors are not anticipated since they must be recorded to allocate treatment. All observed outcome data will be included in the analyses. As this is an exploratory study, we will focus on reporting the amount of missing data. We will report the number (%) of participants withdrawing fully from the trial and lost to follow-up by treatment arm. Reasons for missingness may be important, and these will be tabulated by treatment arm and documented as far as possible. Main analyses will be performed using STATA (StataCorp, College Station, TX, USA).

### Interim analyses {21b}

No interim analyses are planned for the trial.

### Methods for additional analyses (e.g. subgroup analyses) {20b}

Planned subgroup analyses on the primary outcome will investigate interactions between treatment effects and minimisation factors, with separate estimates and CIs reported for these subgroups of participants. In order to explore potential mechanism of action, exploratory analyses will estimate the effect of treatment received on the foetal growth outcomes.

### Methods in analysis to handle protocol non-adherence and any statistical methods to handle missing data {20c}

Every effort will be made to reduce missing data through prospective data monitoring. Level of missing data will be reported for all primary and secondary outcomes.

### Plans to give access to the full protocol, participant-level data and statistical code {31c}

Anonymised data and all statistical code will be made available on request to the chief investigator.

## Oversight and monitoring

### Composition of the coordinating centre and trial steering committee {5d}

The trial is coordinated by the Maternal and Fetal Health Research Centre. All aspects of the conduct and progress of the trial and the day-to-day running of the trial are monitored by the Trial Management Group (TMG). The TMG meets monthly and includes the chief investigator, project manager and trial research midwives. The TMG reports to the Trial Oversight Committee (TOC). The TOC meets at least annually, and as required depending on the needs of the trial, and is responsible for providing overall oversight of the trial, including practical aspects of the trial and ensuring the trial is run safely for the participants. TOC members include an independent chair, two other independent members, a PPI representative, the trial statisticians, the project manager and the CI. The TOC has responsibility for overseeing the conduct of the trial and reviewing the recruitment rate, the baseline characteristics of the trial participants and rates of adverse outcomes. TOC reports are presented to the TOC per arm but not labelled by allocation.

### Adverse event reporting and harms {22}

Adverse events are collected for MIMICH study participants and their babies, from consent (from birth for babies) up to primary discharge after birth. AEs are commonly encountered in this population of pregnant women as a part of the clinical condition of pregnancy and diabetes in pregnancy. As the safety profile of metformin is well-characterised, a strategy of targeted reporting of AEs is used without affecting the safety of participants. Adverse events are separated into expected AEs, which are recorded on the case note review but not reported, and those that are reportable.

The following are considered expected in this population of pregnant women or a result of the usual clinical care and, as such, will be recorded in the participant’s medical record (including where a woman offers information to a research team) but not reported as (S)AEs:

#### Maternal events

- Intolerance to metformin (usual care arm)
- Episodes of hypoglycaemia (not requiring hospitalisation)
- Admission in active labour
- Admission for cervical ripening or induction of labour
- Admission for caesarean section
- Admission for assessment for suspected foetal compromise, including poor growth, or reduced foetal movements
- Admission for the treatment of infection/sepsis
- Admission for monitoring for diabetes, hypertension or pre-eclampsia, antepartum haemorrhage, suspected preterm labour, pre-labour rupture of the membranes or other reasons for monitoring
- Admission for psychiatric or social reasons
- Admission for unstable lie or external cephalic version
- Admission for postpartum complications
- Known complications of pregnancy that are collected for every woman as part of outcome collection (including, but not limited to, pre-eclampsia, postpartum haemorrhage, postpartum haemorrhage requiring transfusion or hysterectomy)
- Any adverse event that is expected during pregnancy including but not limited to tiredness, constipation and back pain. Maternal undesirable effects of the allocated medication regime will be recorded at each trial visit

#### Foetal and neonatal events

Known foetal and neonatal complications of pregnancy that are collected for every infant as part of outcome collection, including, but not limited to:

- Neonatal unit admission
- Miscarriage
- Preterm delivery (< 37 completed weeks’ gestation)
- Neonatal complications (including but not limited to hypoglycaemia, seizures, encephalopathy, need for respiratory support, sepsis, intraventricular haemorrhage, confirmed infection, necrotising enterocolitis, retinopathy of prematurity, congenital anomaly, intraventricular haemorrhage)

For the purposes of this trial and its follow-up (MIMICH II), congenital abnormalities will be considered AEs but not SAEs. As the trial treatment for MIMICH is already licensed and given as or part of standard treatment in this patient population, it is not considered likely that either treatment arm will cause or contribute to a congenital anomaly over and above the risk associated with maternal diabetes.

### Frequency and plans for auditing trial conduct {23}

The central trial team is responsible for monitoring recruitment, providing study-related training and monitoring the quality of the data collected. The site research team is responsible for undertaking all trial-related activities at their recruiting site. The MIMICH trial has been classed as a type A, low-risk trial. This is comparable to the risk of standard medical care as both treatments are routinely used in clinical practice.

Given the low-risk nature of this study, central monitoring is routine, and onsite monitoring is triggered, or as required, as documented in the sponsor/quality management group-approved monitoring plan.

The central trial team are in regular contact with the site research team to check on progress and address any queries. Trial staff regularly check the quality of the data collected for compliance with the protocol, data consistency and completeness. The study data manager sends sites data clarification queries requesting missing data or clarification of inconsistencies or discrepancies. Additional onsite monitoring visits would be triggered, for example by poor electronic case report forms return. The PI must permit trial-related monitoring, audits, ethical review and regulatory inspection(s) at their site, providing direct access to sources.

### Plans for communicating important protocol amendments to relevant parties (e.g. trial participants, ethical committees) {25}

Protocol modifications would be communicated to the appropriate regulatory bodies (Research Ethics Committee (REC) and Health Research Authority (HRA)) and following approval communicated by the sponsor to each site (inclusive of the research management office and local research team). As this is a single-site study, the trial team are in regular contact with the sponsor to address any concerns or issues encountered.

### Dissemination plans {31a}

Trial results will be communicated to healthcare providers and the scientific community via peer-reviewed journals, presentations at national and international conferences. Furthermore, the findings will be disseminated to wider stakeholders via diabetes in pregnancy support groups and charities. Study findings will also be emailed to participants via a lay summary.

## Discussion

Given the uncertainty regarding the potential benefits (improvement in maternal metabolic health and reduction in hypertensive disease) but potential negative effects on placental function (reduction in foetal growth and antiproliferative cellular actions), a trial of metformin in women with hyperglycaemia but who also have risk factors for placental disease is urgently needed. No previous studies have investigated the effect of metformin on placental function, foetal growth and maternal cardiometabolic health in women with hyperglycaemia and concurrent risk factors for placental disease.

The MIMICH trial has been designed to run alongside routine antenatal visits to allow for efficient delivery within standard care, as trial visits and routine care will take place simultaneously. In order to optimise the normalisation of glucose levels for women with diabetes in pregnancy, we are conducting an open-label trial. For this reason, we have chosen an objective measure of foetal growth velocity, which incorporates foetal thigh volume analysed offline by data analysts blinded to treatment allocation. Derivation of the primary outcome after the birth of the baby will therefore reduce bias.

In summary, MIMICH is an open-label, randomised, controlled trial investigating the effect of metformin, primarily on foetal growth, in pregnancies complicated by diabetes for patients with an increased risk of placental disease. It is powered to provide a robust comparison of foetal growth velocity in participants receiving usual treatment for maternal diabetes and those in whom metformin is withheld. We aim to inform future guidance regarding the utility of metformin treatment in this high-risk group. MIMICH II will complement the findings of MIMICH through a prospective observational study, with the primary aim of determining the longer-term impacts of metformin on early postnatal growth and metabolic function in infants born to women with poor cardiometabolic health.

## Trial status

The current version of the MIMICH protocol is version 1.5, 27.06.2022. The current version of the MIMICH II protocol is v2.0, 13.03.2023. The MIMICH trial received approval from REC and HRA on 14.07.2021. The trial opened to recruitment in October 2021, with the first participant recruited on 02.11.2021.

MIMICH II received REC and HRA approval on 01.02.2022, and the trial opened to recruitment on 21.04.2022.

Recruitment to MIMICH is expected to be completed by March 2025. Recruitment to MIMICH II will continue until all MIMICH participants have completed the pregnancy.

## Supplementary Information

Supplementary Material 1.

Supplementary Material 2.

Supplementary Material 3.

## Data Availability

Individuals with access to the full dataset will include the project manager. The chief investigator (CI) will have access to the full dataset after the database lock. Manchester University, the CI’s institution, will be the overall owner of the study data. Site investigators will not have access to the full data set and must not use, disseminate or publish any trial data without the prior written consent of TSC. Site-specific data will be provided to site PIs at the end of the study. Requests for the dataset from appropriate academic parties will be considered by the chief investigator following the data-sharing policies of Manchester University and St Mary’s Hospital Manchester Foundation Trust, with input from the co-investigator group where applicable.

## Abbreviations

AE: Adverse event
APR: Annual progress report
CI: Chief investigator
CTA: Clinical trial authorisation
CTIMP: Clinical trial of investigational medicinal product
CTM: Clinical trials manager
DIBD: Development International Birth Date
eCRF: Electronic case report forms
EFW: Estimated foetal weight
GDM: Gestational diabetes
GCP: Good Clinical Practice
GPRI: Growth Potential Realisation Index
HBGM: Home blood glucose monitoring
HOMA-IR: Homeostatic model assessment of insulin resistance
HRA: Health Research Authority
ICF: Informed consent form
ISF: Investigator site file
ITT: Intention to treat
MHRA: Medicines and Healthcare products Regulatory Agency
NICE: The National Institute for Health and Care Excellence
PI: Principal investigator
PIS: Participant information sheet
PPIE: Public and patient involvement and engagement
R&D: Research and development
RCT: Randomised control trial
REC: Research Ethics Committee
SAE: Serious adverse event
SAR: Serious adverse reaction
SGA: Small for gestational age
SmPC: Summary of product characteristics
TMG: Trial Management Group
TSC: Trial Steering Committee
TOC: Trial Oversight Committee
QALYs: Quality-adjusted life years
UoM: University of Manchester
USM: Urgent safety measure
