# Supplementary material for: Metformin Impact on Maternal and Infant Cardiometabolic Health (MIMICH), an open-label randomised controlled trial, and Metformin Impact on Maternal and Cardiometabolic Health After Pregnancy (MIMICH II)
Source: Trials. 2025 Oct 17;26:423. doi: 10.1186/s13063-025-09154-5 (PMC12535036; doi:10.1186/s13063-025-09154-5)
Supplement: Supplementary file 1 — Supplementary Material 1. [file 13063_2025_9154_MOESM1_ESM.docx]

Appendix A Study Instruments

| Outcomes | Instrument |
| --- | --- |
| Fetal biometry and 3D volumetric measurement | GE Healthcare Voluson (E8, E10, S10) |
| Patient satisfaction | Study questionnaire, appendix B |
| Continuous glucose monitoring | FreeStyle Libre* |
| Capillary glucose measurement | Contour Next* |
| Hba1c | BioRad HPLC |
| Insulin | Roche immunoassay |
| Lipid profile | Homogeneous enzymatic colorimetric test, Roche |
| Triglycerides | Enzymatic colorimetric test, Roche |
| sflt/PlGF ratio | Roche immunoassay |
| PlGF | Quidel Triage PlGF Test, QuidelOrtho |

*Methods of glucose monitoring routinely used in the organisation, participants in the study may be established on other clinically validated methods.
