# Supplementary material for: Metformin Impact on Maternal and Infant Cardiometabolic Health (MIMICH), an open-label randomised controlled trial, and Metformin Impact on Maternal and Cardiometabolic Health After Pregnancy (MIMICH II)
Source: Trials. 2025 Oct 17;26:423. doi: 10.1186/s13063-025-09154-5 (PMC12535036; doi:10.1186/s13063-025-09154-5)
Supplement: Supplementary file 2 — Supplementary Material 2. [file 13063_2025_9154_MOESM2_ESM.pdf]

Acceptability Questionnaire

Study ID ______________________

Initials **************************** [initial]

Please complete the following questions

1. Were you happy to participate in the study?
   - ◯ Yes
   - ◯ No

If no, please could you tell us why? ______________________

2. Which group were you randomised to?
   - ◯ No metformin
   - ◯ Meformin

3. When you started the study, how did you feel about your allocation?
   - ◯ a) Not satisfied
   - ◯ b) Satisfied
   - ◯ c) Not sure

4. How do you feel about your allocation now?
   - ◯ a) Not satisfied
   - ◯ b) Satisfied
   - ◯ c) Not sure

Comments ______________________

The following questions relate to the medication and treatment you received as part of the study

5. How often did you forget to take your insulin injections?
   - ◯ a) I was not prescribed insulin injections
   - ◯ b) Never or rarely
   - ◯ c) 1-3 times/wk
   - ◯ d) 4-6 times/wk
   - ◯ e) >6 times/wk

6. How often did you forget to take metformin tablets?
   - ◯ a) I was not prescribed metformin tablets
   - ◯ b) Never or rarely
   - ◯ c) 1-3 times/wk
   - ◯ d) 4-6 times/wk
   - ◯ e) >6 times/wk

7. Which part of your diabetes treatment was the easiest?
   - ◯ a) Doing finger prick tests
   - ◯ b) Being careful with diet
   - ◯ c) Taking meformin
   - ◯ d) Using insulin injections

8. Which part of your diabetes treatment was the hardest?
   - ◯ a) Doing finger prick tests
   - ◯ b) Being careful with diet
   - ◯ c) Taking medication
   - ◯ d) Using insulin injections

Do you have any other comments?

____
